# Supplementation with honeysuckle extract improves growth performance, immune performance, gut morphology, and cecal microbes in geese

**DOI:** 10.3389/fvets.2022.1006318

**Published:** 2022-11-03

**Authors:** Guangquan Li, Xianze Wang, Yi Liu, Cui Wang, Yunzhou Yang, Shaoming Gong, Lihui Zhu, Daqian He, Huiying Wang

**Affiliations:** Institute of Animal Husbandry and Veterinary Science, Shanghai Academy of Agricultural Sciences, Shanghai, China

**Keywords:** goose, growth performance, immune, intestinal morphology, cecal microbiome

## Abstract

The study aimed to investigate the effects of honeysuckle extract (HE) on growth performance, serum biochemical indexes, immune organ indexes, gut morphology, and gut microbes in geese. A total of 180 28-day-old Holdobaki geese were randomly divided into three groups. Each group contained 6 replicates (10 geese, with 5 males and 5 females). The BD group was fed the basal diet, the HE1 group was fed the basal diet supplemented with 1 g/kg of HE, and the HE2 group was fed the basal diet supplemented with 2 g/kg of HE. The experiment lasted for 42 days. The results showed that, compared with the BD group, the average daily gain (ADG) of the HE1 and HE2 groups tended to increase (0.05 < *P* < 0.10), but the average daily feed intake (ADFI) and final body weight (BW) did not differ significantly, and the feed/gain ratio (F/G) was significantly lower (*P* < 0.01). The bursa index and the thymus index tended to increase (0.05 < *P* < 0.10), and serum immunoglobulin A (IgA) and immunoglobulin G (IgG) levels increased significantly (*P* < 0.05). In the HE1 and HE2 groups, the crypt depth (CD) in the jejunum tended to decrease (0.05 < *P* < 0.10), and the villus height/crypt depth ratio (V/C) increased significantly in the jejunum and the ileum (*P* < 0.05). According to 16sRNA microbial community diversity analysis, *Firmicutes, Bacteroidetes, Proteobacteria*, and *Actinobacteria* were the dominant phyla. The abundance of *Firmicutes* was significantly decreased (*P* < 0.01), while that of *Bacteroidetes* was significantly increased (*P* < 0.01), in the HE1 and HE2 groups compared with the BD group. *Bacteroides barnesiae, Subdoligranulum variabile, Bacteroides plebeius*, and *Faecalibacterium prausnitzii* were the dominant species, and the abundance of *B. plebeius* and *F. prausnitzii* was significantly increased (*P* < 0.05). According to the LEfSe analysis, BD enriched *g_Dorea* and *g_Dehalobacterium*; HE1 enriched g_*Faecalibacterium, g_Dialister, g_Prevotella, g_Megamonas, g_Phascolarctobacterium, g_Paraprevotella, g_Anaerostipes, g_Staphylococcus, g_Odoribacter, g_Succinivibrio*, and *g_Sutterella*; and HE2 enriched g_*Parabacteroides, g_Olsenella, g_human*, and *g_Rikenella*. According to the Spearman correlation analysis, *Bacteroides plebeius* was positively correlated with final BW, ADG, IgA, IgG, VH (ileum), and V/C (ileum) and was negatively correlated with F/G and CD (ileum); *Ruminococcus gnavus* was negatively correlated with final BW, ADG, IgA, and IgG. HE supplementation at 1 g/kg improved growth performance, immune performance, gut morphology, and cecal microbes.

## Introduction

Intensive poultry farming causes environmental pollution problems, which will affect poultry immunity, resulting in higher mortality and economic losses. Supplementing antibiotics can improve poultry performance and reduce mortality. Therefore, antibiotics will be supplemented during the breeding process to enhance immunity. However, the abuse of antibiotics will lead to the problem of drug resistance and antibiotic residues. As using antibiotics will affect the health of humans and animals and destroy the microbial ecosystem, many countries have completely banned the use of antibiotics in animal husbandry (1, 2). Therefore, it has become a trend to replace antibiotics with natural and environment-friendly alternative substances. In the current research, organic acids, plant extracts, yeasts, and probiotics are potential alternative products (3, 4). Plant extracts are widely used in agriculture and play an important role in sustainable agriculture (5). Honeysuckle is a traditional medicine that has been widely used since ancient China. It has high nutrition and is rich in carbohydrates, protein, crude fat, crude fiber, vitamins, minerals, and other nutrients; it also contains phenols, ketones, and other substances, which have various effects, such as lowering blood sugar, improving anti-oxidation, and improving immunity (6–8). However, honeysuckle also contains anti-nutritional factors such as tannins, and excessive supplementation will be detrimental to animals. Separating and purifying honeysuckle and processing HE can effectively preserve the beneficial substances while removing harmful substances. Meng et al. found that supplementing HE could reduce the mRNA expression of fat synthesis-related genes, strengthen the intestinal mucosal barrier of grass carp, improve lipid metabolism and immune function, and reshape the intestinal flora (9, 10). Zhao et al. found that supplementing HE during the perinatal period improved dry matter intake, lactation capacity, anti-inflammatory properties, and antioxidant capacity of dairy cows (11). Its effect on geese has not been reported so far. Therefore, in this experiment, 28-day-old Holdobaki geese were used as a sample, and different proportions of HE were supplemented into the diet to explore its effects on growth performance, immune performance, intestinal morphology, and cecal microbes.

## Materials and methods

### Experimental design, diets, and birds

The experimental protocol was approved by the Shanghai Academy of Agricultural Sciences, and the experimental methods and ethics complied with relevant regulations.

Holdobaki goose is an excellent variety cultivated by the Hungarian Holdobaki Goose Co., Ltd. It has the characteristics of fast growth and delicious meat. It is marketed as a product at 60–70 days of age, with an average weight of about 4,500 g. The geese were purchased from Anhui Xiangtiange Goose Industry Professional Cooperative (28 days old, a total of 180 males and females), and the experimental site was Zhuanghang Experimental Station of Shanghai Academy of Agricultural Sciences. Each goose was weighed before the start of the experiment, and its initial average body weight was taken. They were divided into 3 groups, with each group having 6 cages, and the sizes of each cage were 200 cm/200 cm/50 cm. There was no significant difference between the groups. The first group was fed with a basal diet (BD), and the second and third groups were fed with a basal diet supplemented with 1 g/kg of HE (HE1) and 2 g/kg of HE (HE2), respectively (produced by Shaanxi Tangmen Biotechnology Co., Ltd., China). The basal diet formula (Table 1) refers to the nutrient requirements recommended by the NCR Standard (12) and according to the actual design of China's current goose production.

**Table 1:** Ingredients and composition of basal diets (DM basis) %.

**Ingredients, g/kg**		**Nutrition levels^b^, g/kg**	
Corn	58.16	ME(MJ/kg)	2.70
Soybean meal	25.60	Crude protein	19.49
Bran	10.10	Crude fiber	7.00
Soybean oil	1.50	Ca	0.46
Stone power	1.00	Total P	0.32
Methionine	0.18	Methionine	0.43
Threonine	0.09	Lysine	1.056
Lysine	0.37	Threonine	0.742
3%Premix^a^	3.00	Methionine + Cystine	0.688

a One kilogram of the premix contained the following: Fe 100 mg, Cu 8 mg, Mn 120 mg, Zn 100 mg, Se 0.4 mg, Co 1.0 mg, I 0.4 mg, VA 8330 IU, VB1 2.0 mg, VB 2.8 mg, VB6 1.2 mg, VB12 0.03mg, VD3 1440IU,VE 30 IU,biotin 0.2 mg, folic acid 2.0 mg, pantothenic acid 20 mg, niacin acid 40 mg.

b Nutrient levels were all calculated values.

### Growth performance

At the beginning (day 28) and end (day 70) of the experiment, the geese in each cage were weighed, and the average daily weight gain (ADG), average daily feed intake (ADFI), and feed/gain ratio (F/G) of each cage were calculated. The geese were fasted for 8 h before weighing.

### Sample collection

At the end of the experiment, one goose (male) with a weight close to the mean was selected from each cage. Blood was collected from the fin vein and placed in a vacuum blood collection tube. After standing at room temperature for 5 h, centrifugation at 3,000 rpm for 10 min was performed to collect serum. Then, the jugular vein was bled and slaughtered, the abdominal cavity was opened, the bursa, the spleen, and the liver were taken out first, and the adhering fat was removed immediately after weighing and recording, and the immune organ index was calculated (immune organ index = immune organ weight, g/live weight, kg). The jejunum and the ileum were removed, the contents of the digestive tract of the jejunum were gently squeezed out, the residue was rinsed with normal saline, the residual water in the digestive organs was dried with a filter paper, the jejunum and ileum anterior segments were cut (one-fourth of the front end) at approximately 2 cm, and normal saline was used. The contents were rinsed, fixed with 10% formaldehyde, sectioned with paraffin, stained with hematoxylin-eosin, and observed under a light microscope.Villus height (VH) was measured from the tip (with a lamina propria) of the villus to the base (villus-crypt junction), crypt depth (CD) was measured from the villus-crypt junction to the distal limit of the crypt, and villus height/crypt depth ratio (V/C) was calculated. In supplementation, cecal chyme samples were taken into 2 ml EP tubes, which were snap-frozen in liquid nitrogen and stored at 80°C for later use.

### Serum biochemical indicators

The collected serum samples were sent to Shanghai Pinyi Biotechnology Co., LTD to detect transforming total protein (TP), albumin (ALB), glucose (GLU), total cholesterol (CHOL), low-density lipoprotein cholesterol (LDL-C), high-density lipoprotein cholesterol (HDL-C), growth factor-β (TGF-β), interleukin 10 (IL-10), immunoglobulin A (IgA), and immunoglobulin G (IgG).

### 16sRNA microbial community diversity analysis

For 16S rRNA sequencing analysis and data processing, Shanghai Personal Technology Co., Ltd. used gut microbes. Using the method of Divisive Amplicon Denoising Algorithm (13), noise reduction was used to obtain biological sequence ASVs (amplicon sequence variants) that did not contain amplification, sequencing errors, or chimeras. To comprehensively assess the α-diversity of microbial communities, we used the Chao1 index and observed species indices to characterize richness, Shannon and Simpson indices to characterize diversity, Faith's PD index to characterize evolution-based diversity, and Pielou's evenness index to characterize evenness.

### Statistical analysis

The experimental data were analyzed using a one-way ANOVA in SPSS software (SPSS 26.0, Abacus Concepts, Berkeley, CA, United States), and Duncan's multiple comparison method was used to test whether there were significant differences between groups. The results were expressed as the mean and standard error of the mean (SEM), with a *Pi-*value < 0.05 indicating a significant difference. The Spearman correlation analysis method was used to determine the relationship between the cecal microbial communities and the measured parameters.

## Results

### Growth performance

The growth performance is shown in Table 2. Compared with the BD group, the final BW of the HE1 and HE2 groups increased, but the difference was not significant (*P* = 0.178). The ADG increased (*P* = 0.076), and the ADFI decreased (*P* = 0.168). The F/G ratio increased significantly (*P* = 0.001).

**Table 2:** Effect of HE on growth performance^a^.

	**Treatment**				
**Items**	**BD**	**HE1**	**HE2**	**SEM**	***P*** **-value**
Initial BW, g	2000.25	1982.65	1976.48	19.187	0.873
Final BW, g	4305.70	4457.52	4459.77	38.758	0.178
ADG,g/d	47.05	50.51	50.68	0.738	0.076
ADFI,g/d	258.06^b^	259.35^a^	259.52^a^	0.344	0.168
F/G	5.48^a^	5.13^b^	5.12^b^	0.051	0.001

Different lowercase letters with the same column date meant significant difference (P ≤ 0.05).

a Each value represents the mean of 6 replicate pens.

The a and b indicates the content of the form indicate that the difference is significant.

### Immune organ indexes

The immune organ index is shown in Table 3. There was no significant difference in the spleen index, but the thymus index and the bursa of the Fabricius index increased in the HE2 group (*P* = 0.052).

**Table 3:** Effect of HE on immune organ index^a^.

	**Treatment**				
**Items**	**BD**	**HE1**	**HE2**	**SEM**	***P*** **-value**
Thymus index	0.73	0.89	0.80	0.032	0.054
Spleen Index	0.59	0.66	0.58	0.054	0.822
Bursa of Fabricius index	0.43	0.54	0.56	0.027	0.052

Different lowercase letters with the same column date meant significant difference (P ≤ 0.05).

^a^Each value represents the mean of 6 replicate pens.

### Serum immune index

The effect of HE on goose serum immune indexes is shown in Table 4. The serum IgA and IgG were significantly increased in the HE1 and HE2 groups compared with the BD group (*P* = 0.015, *P* = 0.037).

**Table 4:** Effect of HE on serum biochemical indexes^a^.

	**Treatment**				
**Items**	**BD**	**HE1**	**HE2**	**SEM**	***P*** **-value**
TP, g/L	47.45	45.50	43.45	0.961	0.247
ALB, g/L	11.30	10.90	10.77	0.173	0.452
ALP, U/L	207.17	201.33	201.17	10.040	0.966
GLU, mmol/L	10.88	11.18	11.73	0.235	0.346
CHOL, mmol/L	3.52	3.77	3.36	0.102	0.287
HDL-C, mmol/L	2.01	2.23	1.95	0.649	0.174
LDL-C, mmol/L	1.17	1.03	1.17	0.053	0.512
TGF-β, pg/ml	226.31	170.03	360.33	48.546	0.273
IL-10, pg/ml	140.79	107.97	141.50	9.865	0.301
IgA, mg/ml	12.84^b^	20.45^a^	21.63^a^	1.443	0.015
IgG, mg/ml	32.50^b^	46.94^a^	52.68^a^	3.455	0.037

Different lowercase letters with the same column date meant significant difference (P ≤ 0.05).

a Each value represents the mean of 6 replicate pens.

The a and b indicates the content of the form indicate that the difference is significant.

### Intestinal morphology

Table 5 shows that, compared with the BD group, there was no difference in VH between the HE1 and HE2 groups (*P* = 0.723) in the jejunum, the CD had a decreasing trend in the HE1 and HE2 groups (*P* = 0.072), and the V/C ratio of the HE1 and HE2 groups significantly improved (*P* = 0.021). In the ileum, compared with the BD, there was no difference in VH between the groups (*P* = 0.440), the CD had a decreasing trend in the HE1 and HE2 groups (*P* = 0.157), and the V/C ratio of the HE1 and HE2 groups significantly improved (*P* = 0.034).

**Table 5:** Effect of HE on intestinal morphometry^a^.

	**Treatment**				
**Items**	**BD**	**HE1**	**HE2**	**SEM**	***P*** **-value**
Jejunum					
VH,um	499.30	503.21	509.96	5.441	0.723
CD,um	103.90	93.89	94.93	1.960	0.072
V/C	5.12^c^	5.51^b^	5.98^a^	0.128	0.021
Ileum					
VH,um	444.55	452.92	457.62	4.203	0.440
CD,um	92.97	87.56	87.09	1.387	0.157
V/C	4.94^b^	5.67^a^	5.42^a^	0.205	0.034

Different lowercase letters with the same column date meant significant difference (P ≤ 0.05).

a Each value represents the mean of 6 replicate pens.

The a and b indicates the content of the form indicate that the difference is significant.

### Gut microbial diversity and composition

**Figure 1:**
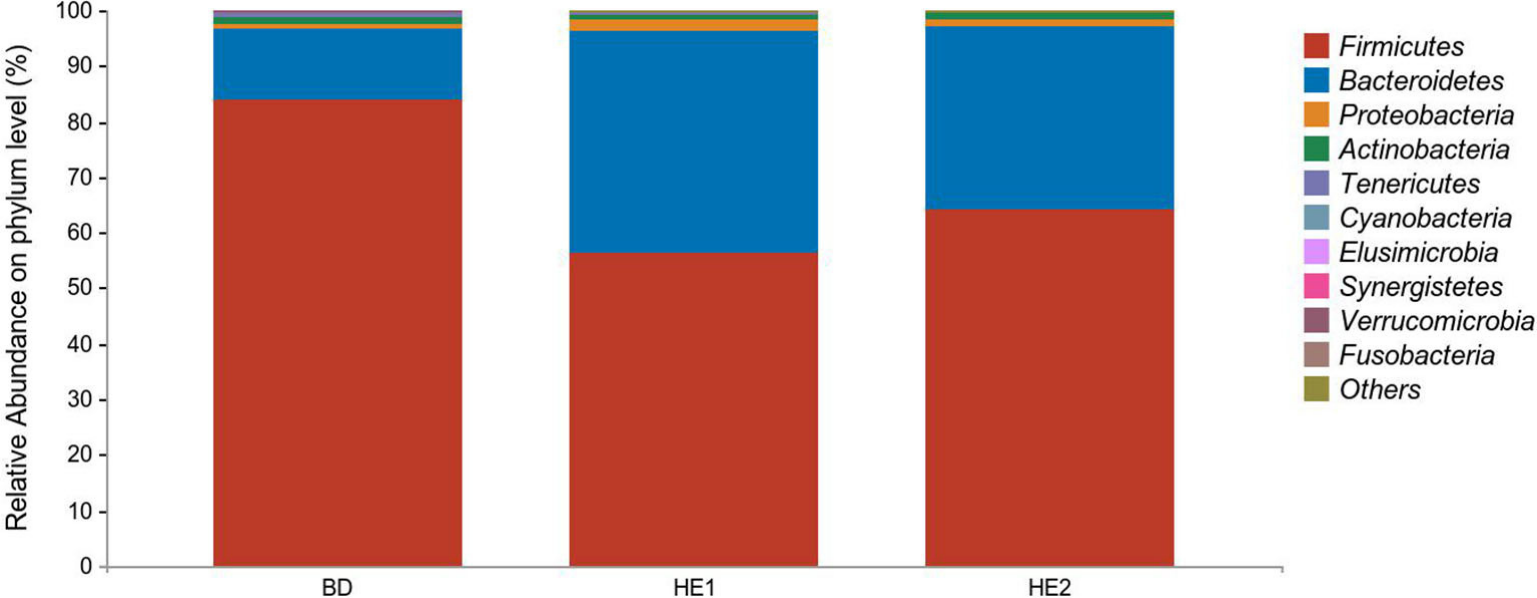
Relative abundance at the phylum level between groups.

**Figure 2:**
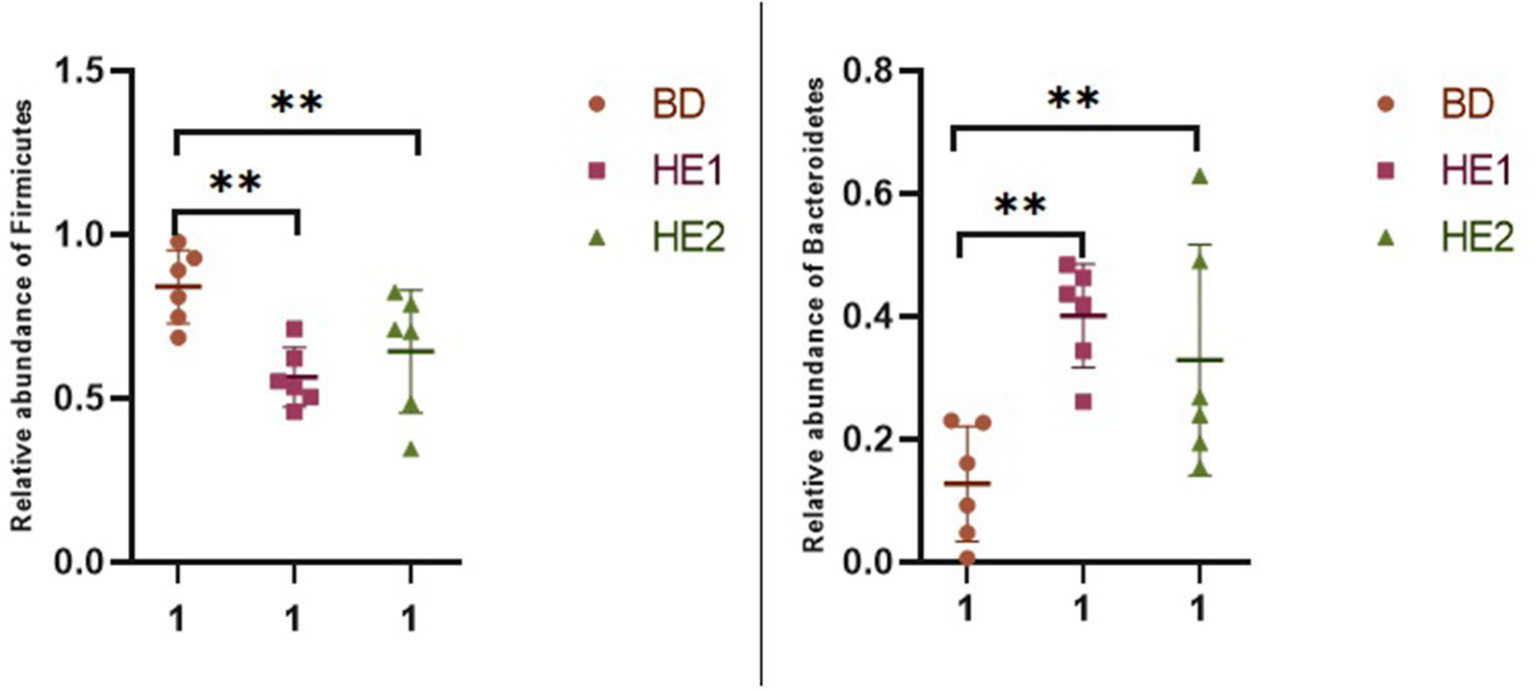
Scatter plot of the relative abundance of significantly different phylum in each group.

**Figure 3:**
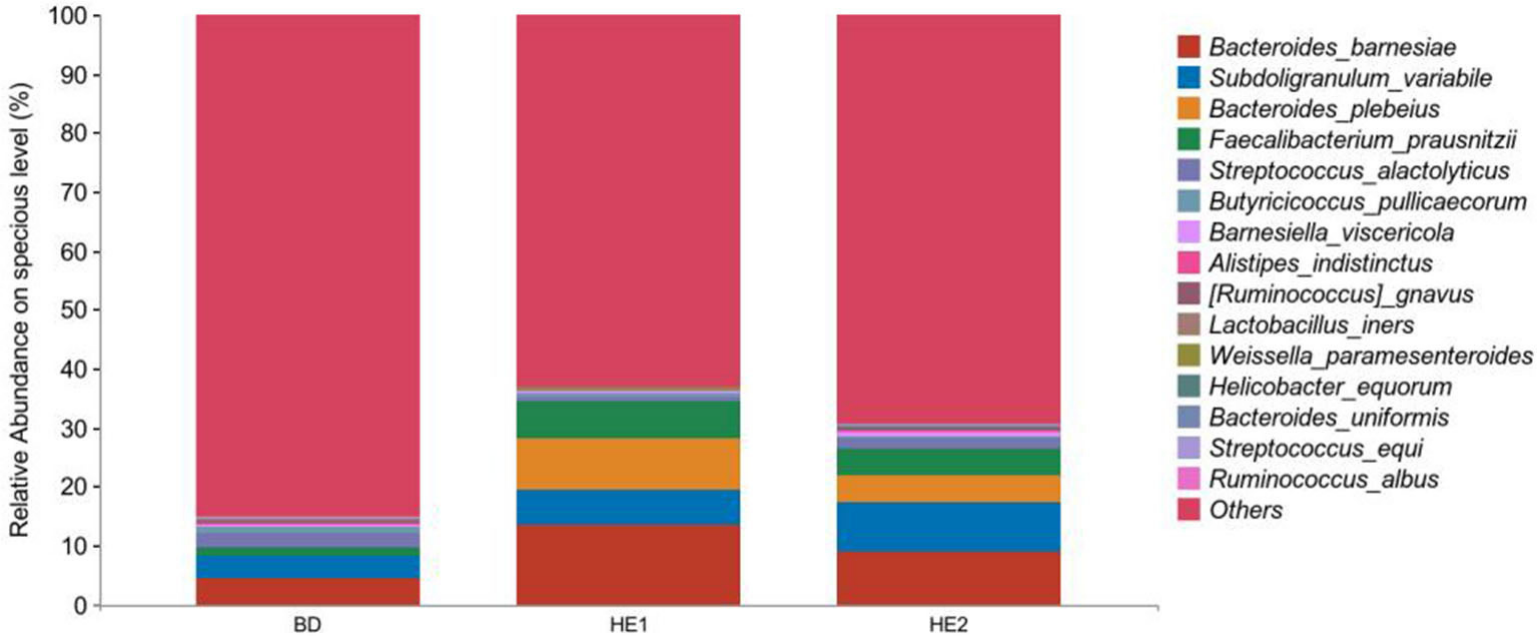
Relative abundance at the specious level between groups.

**Figure 4:**
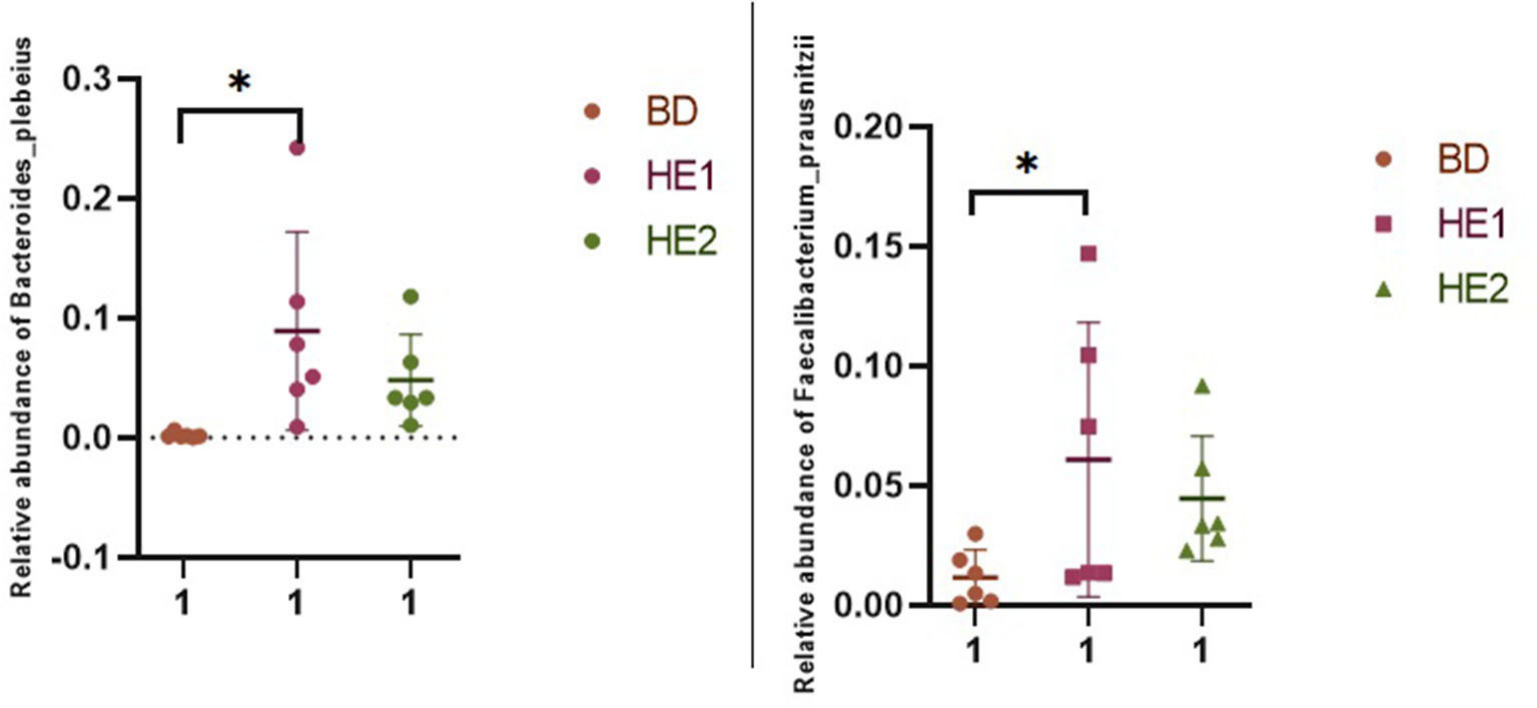
Scatter plot of the relative abundance of significantly different specious in each group.

**Figure 5:**
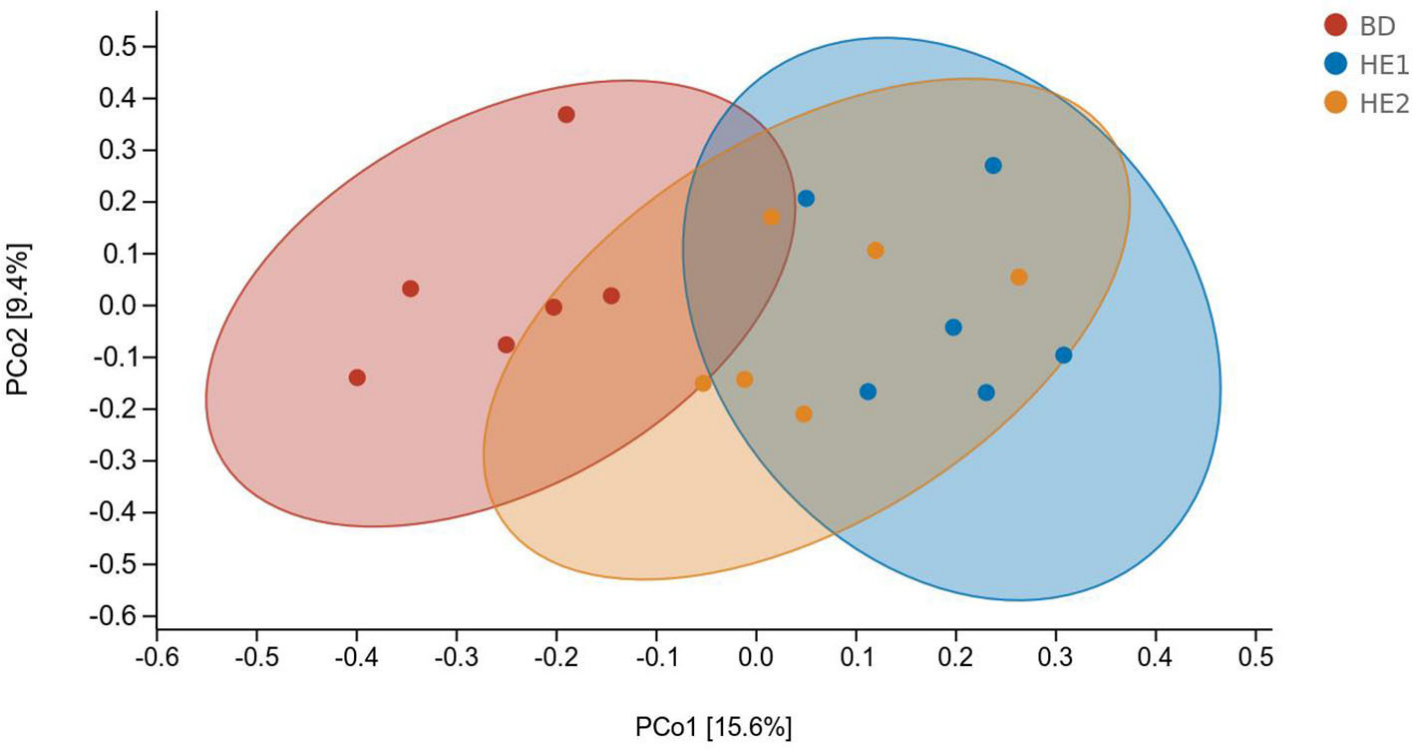
The PCoA based on the Bray-Curtis distance.

**Figure 6:**
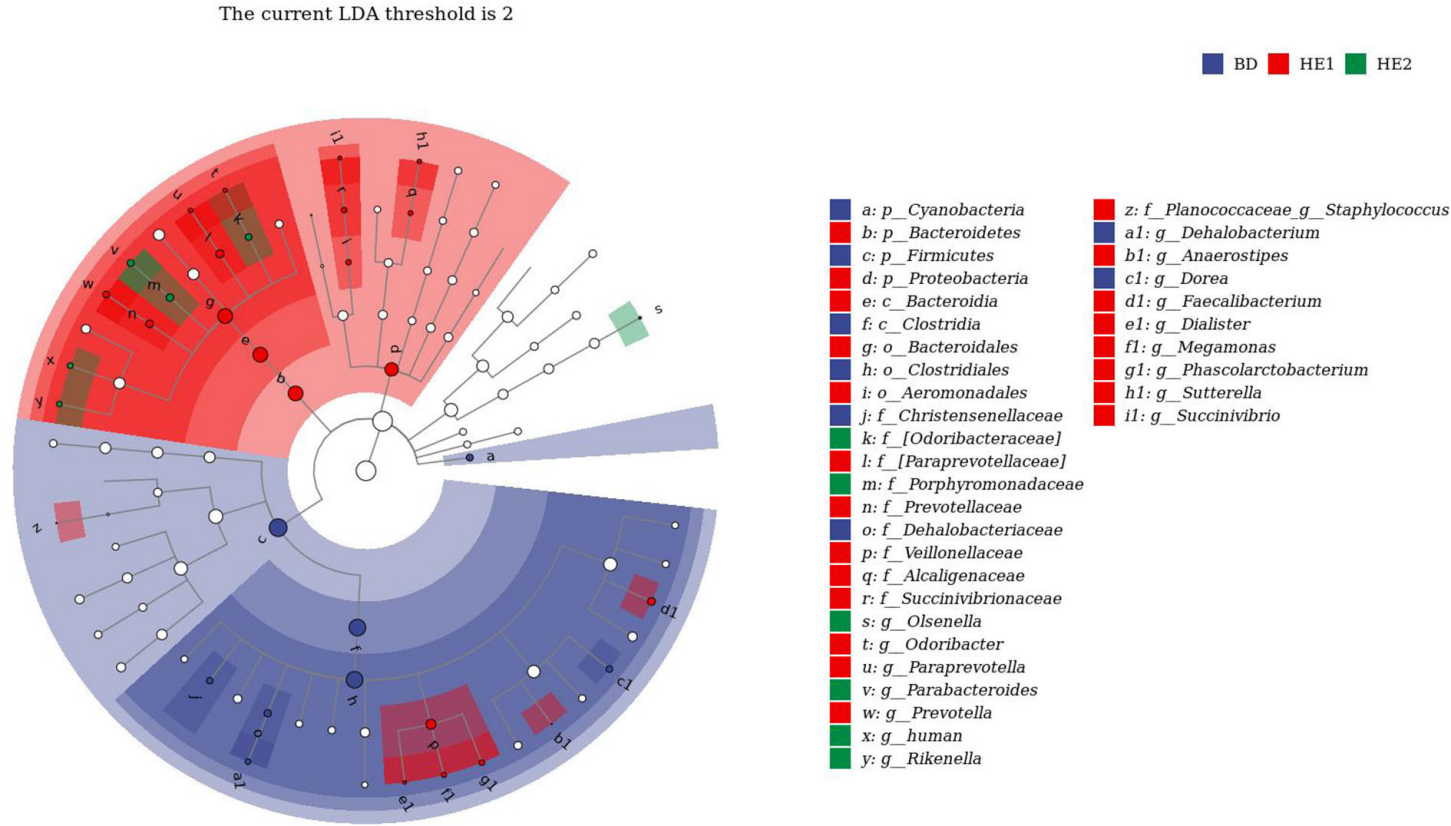
LEfSe analysis of differential microorganisms.

α-Diversity refers to the indicators of richness, diversity, and evenness of species in a locally homogeneous habitat. The differences in each index of α-diversity are shown in Table 6, and there is no significant difference in groups. Figure 1 shows the average relative abundance at the phylum level. *Firmicutes, Bacteroidetes, Proteobacteria*, and *Actinobacteria* were the dominant species in the cecum. Figure 2 shows some phyla with significant differences between groups. Compared with the BD group, we found that the abundance of *Firmicutes* was significantly decreased (*P* < 0.01) while the abundance of *Bacteroidetes* was significantly increased (*P* < 0.01) in the HE1 and HE2 groups. Figure 3 shows the average relative abundance at the species level. *Bacteroides barnesiae, Subdoligranulum variabile, Bacteroides plebeius*, and *Faecalibacterium prausnitzii* were the dominant species in the cecum. Compared with the BD group, we found that the abundance of *Bacteroides plebeius* and *Faecalibacterium prausnitzii* were significantly increased (*P* < 0.05) in the HE1 group (Figure 4). Figure 5 shows the PCoA based on the Bray-Curtis distance. The HE1 and HE2 groups were gathered in a certain area and had a relatively obvious distance from the BD. The PCo1 value is 15.6%, and the PCo1 value is 9.4%. We used LEfSe to find out which genus levels were different in each group. As shown in Figure 6, BD enriched *g_Dorea* and *g_Dehalobacterium*; HE1 enriched g_*Faecalibacterium, g_Dialister, g_Prevotella, g_Megamonas, g_Phascolarctobacterium, g_Paraprevotella, g_Anaerostipes, g_Staphylococcus, g_Odoribacter, g_Succinivibrio*, and *g_Sutterella*; and HE2 enriched g_ *Parabacteroides, g_Olsenella, g_human*, and *g_Rikenella*.

**Table 6:** Effect of HE on α-diversity^a^.

	**Treatment**				
**Items**	**BD**	**HE1**	**HE2**	**SEM**	***P*** **-value**
Chao1	2646.26	2901.59	2890.06	119.27	0.642
Observed_species	2397.80	2674.15	2592.62	110.59	0.606
Shannon	8.19	8.62	8.65	0.161	0.460
Simpson	0.97	0.98	0.99	0.004	0.356
Pielou_e	0.73	0.76	0.76	0.011	0.444
Faith_pd	130.11	148.46	141.11	4.672	0.287
Goods_coverage	0.99	0.99	0.99	0.001	0.646

Different lowercase letters with the same column date meant significant difference (P ≤ 0.05).

^a^Each value represents the mean of 6 replicate pens.

### Relationship between gut microbiota and main parameters

**Figure 7:**
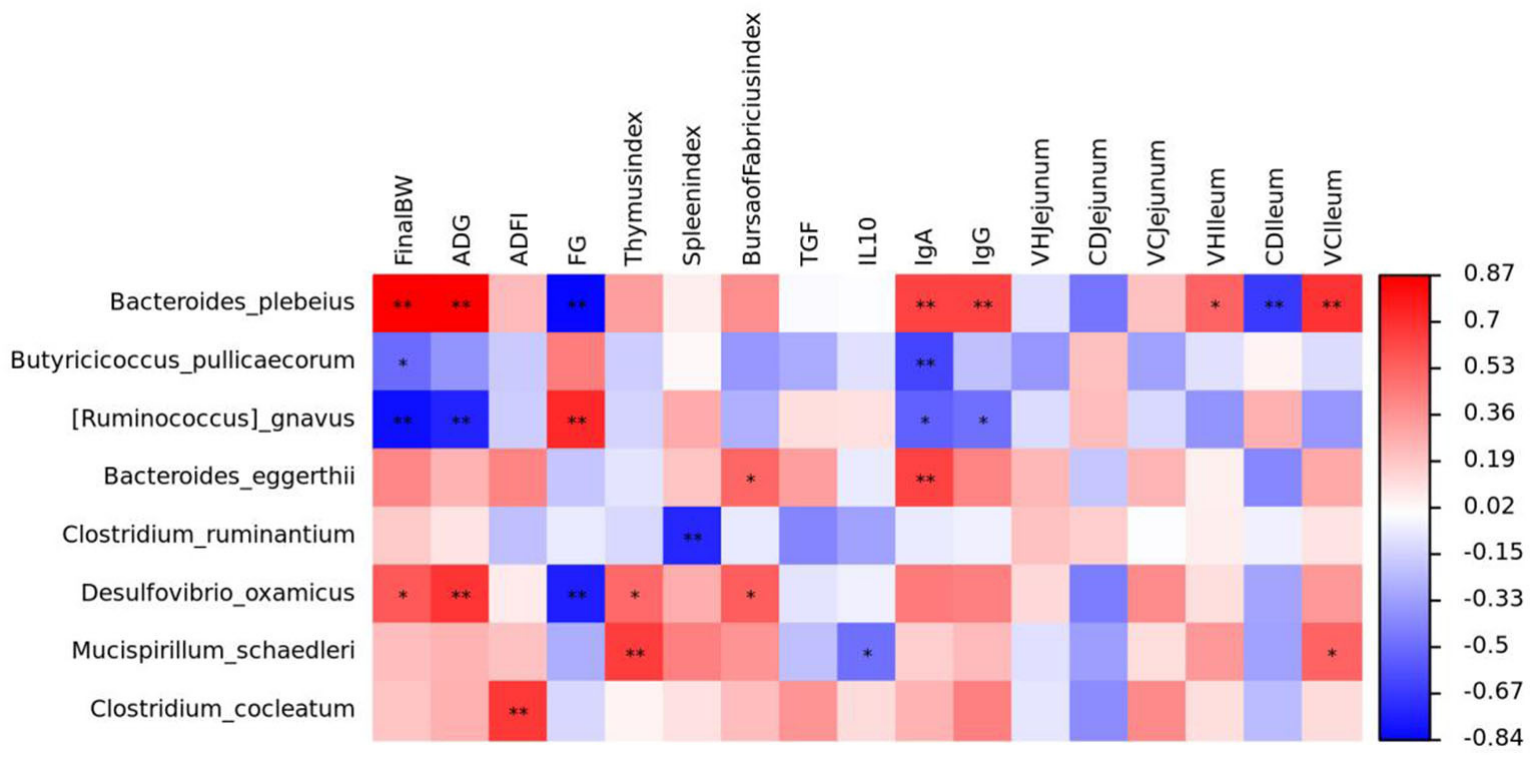
Relationship between gut microbiota and main parameters.

We used Spearman's correlation heatmaps to predict the relationship between the species and key parameters (Figure 7). *Bacteroides plebeius* was positively correlated with final BW, ADG, IgA, IgG, VH (ileum), and V/C (ileum) and was negatively correlated with F/G and CD (ileum); *Butyricicoccus pullicaecorum* was negatively correlated with final BW and IgA; *Ruminococcus gnavus* was negatively correlated with final BW, ADG, IgA, and IgG; *Bacteroides eggtherii* was positively correlated with IgA and the bursa of the Fabricius index; *Clostridium ruminantium* was negatively correlated with the spleen index; *Desulfovibrio oxamicus* was positively correlated with final BW, ADG, the bursa of Fabricius index, and the thymus index and was negatively correlated with F/G; *Mucisoirillum schaedleri* was positively correlated with the thymus index and V/C (ileum) and was negatively correlated with IL-10; *Clostridium cocleatum was* negatively correlated with ADFI.

## Discussion

Honeysuckle contains a variety of organic acids and polyphenols that can increase the secretion of enzymes in the stomach, improve the activity of digestive enzymes, and promote gastric motility and digestion of fatty foods, thereby enhancing the appetite and feed intake of young animals, promoting weight gain. Supplementing 400 g/day of honeysuckle to sheep diet can significantly increase final weight and average daily gain (14). Liu found that the supplementation of *Scutellaria baicalensis* and HE mixture (0.025 and 0.05%) improved growth performance, nutrient digestibility, and meat quality in finishing pigs (15). J.H. Park found that supplementing 0.2% HE improved broiler performance, blood cells, and meat quality (16). Chlorogenic acid (CGA) is a component of HE. Supplementation of 1 g/kg of CGA improved growth performance, immune function, antioxidant status, and intestinal barrier function in coccidial-infected broilers (17). Dietary CGA supplementation at 1 g/kg improved growth performance and quality and oxidative statuses of meat in pigs subjected or not to oxidative stress induced by dietary oxidized oil (18). A combination of CGA and bamboo charcoal particles can effectively improve the performance and husbandry environment of broilers (19). In this experiment, we found that the supplementation of 1 g/kg and 2 g/kg of HE to the goose diet can significantly improve the F/G ratio and the ADFI, and the ADG has a tendency to increase. At the same time, we found that the supplementation of 1 g/kg and 2 g/kg has a tendency to reduce the CD of the ileum and the jejunum and can significantly increase the V/C ratio of the ileum and the jejunum. This may be related to the fact that the supplementation of HE increased the V/C ratio, which in turn increased the daily gain and F/G ratio. Several studies found that there was a positive correlation between production performance and the V/C ratio (20–22).

Previous research showed that plant extracts can improve the immune organ index and the serum immune performance of animals (23, 24). The study found that HE improves cellular and innate immunity in immunosuppressed mice and promotes the secretion of immune-related cytokines through the iNOS-related signaling pathways, thereby exerting immunomodulatory activity (25). HE can modulate immunity by inhibiting the apoptosis of mouse lymphocytes (26). CGA at 215 μg/egg showed the significant potential of anti-infectious bursal disease virus (27). The above results are similar to the results of this experiment. We found that the supplementation of HE has a tendency to increase the bursa index and can significantly increase the serum IgA and IgG levels. It shows that HE can improve the immune performance of geese.

The normal microbial flora in the animal gastrointestinal tract plays an important role in maintaining animal health and normal physiological state, improving animal body resistance, and inhibiting the colonization of potentially pathogenic bacteria in the animal gastrointestinal tract (28). Bacteria in the microbiota produce a number of different compounds, including vitamins (vitamin K and B vitamins) (29, 30), volatile fatty acids (31), organic acids (lactic acid) (32), and antibacterial compounds (bacteriocins) (33). The microbiome provides protection and nutrition for animals. In the intestines of animals, the intestinal flora is mainly composed of *Firmicutes* and *Bacteroidetes*. *Bacteroidetes* are gram-negative and related to immune regulation. They are composed of lipopolysaccharides and flagellin, which interact with cellular receptors and enhance immune responses through cytokine synthesis (34). In this study, we found that the abundance of *Bacteroidetes* increased after supplementing HE. This may be positively correlated with improved immune properties such as the bursa index and serum IgA and IgG. The *Firmicutes*/*Bacteroidetes* ratio (F/B) is widely believed to play an important role in maintaining normal intestinal homeostasis (35). The high-fat diet causes an increase in the F/B ratio that can be recovered after treatment with plant extracts (36). This may be the reason for the decreased F/B ratio in this experiment. Therefore, we believe that supplementing HE changed the proportion of dominant flora at the cecal phylum level and promoted intestinal health.

Using LEfSe analysis, we found that *Faecalibacterium* was enriched in the HE1 group, and *Faecalibacterium prausnitzii* was significantly increased using a one-way ANOVA. *F. prausnitzii* is an acetate consumer that produces butyrate and bioactive anti-inflammatory molecules such as shikimic and salicylic acids. It improves intestinal inflammation (37, 38) and insulin resistance (39). Late weaning is associated with increased microbial diversity and the abundance of *F. prausnitzii* in the fecal microbiota of piglets (40). Supplementation of *F. prausnitzii* to lactating calves reduces the incidence of diarrhea, improves ADG, and increases weaning weight (41, 42). In this experiment, supplementing 1 g HE can significantly increase the abundance of *F. prausnitzii*.

Through Spearman's analysis of the relationship between the main parameters and the gut microbiome, *Bacteroides plebeius* and *Bacteroides eggtherii* were positively correlated with many growth performance and immune indicators. *Bacteroides* are involved in many important metabolic activities in the human colon, including the fermentation of carbohydrates, the utilization of nitrogenous substances, and the biotransformation of bile acids and other steroids. The main by-products of anaerobic respiration are acetic acid, isovaleric acid, and succinic acid (43). *Bacteroides plebeius is* a key enzyme in initiating the depolymerization of agarose in the human gut (44). The abundance of *Ruminococcus gnavus* is linked to various diseases (45, 46). The adhesin produced by *R. gnavus* in the gut will preferentially bind IgA or IgG, which may be the reason why *R. gnavus* was negatively correlated with IgA or IgG in this experiment.

## Conclusions

Supplementation of honeysuckle extract in the diet can effectively improve growth performance, intestinal morphology, and immune performance by altering gut microbial composition and antioxidant capacity. The best supplementation level is 1 g/kg.

## Data availability statement

The datasets presented in this study can be found in online repositories. The name of the repository and accession number can be found below: NCBI; PRJNA865537.

## Ethics statement

The experimental procedure was approved by the Shanghai Academy of Agricultural Sciences (SAASPZ0522046) and the experimental methods and ethics complied with relevant regulations.

## Author contributions

Writing-original draft: GL. Formal analysis: GL and XW. Investigation: GL, YY, and LZ. Conceptualization: SG, YL, and CW. Project administration: DH. Writing—review, editing, validation, and supervision: HW and DH. Funding acquisition: HW. All authors have read and agreed to the published version of the manuscript.

## Funding

This research was funded by China Agriculture Research System of MOF and MARA, grant number CARS-42-35. Climbing plan of Shanghai Academy of Agricultural Sciences, grant number PG21171 and SAAS Program for Excellent Research Team, grant number 2022-021.

## Conflict of interest

The authors declare that the research was conducted in the absence of any commercial or financial relationships that could be construed as a potential conflict of interest.

## Publisher's note

All claims expressed in this article are solely those of the authors and do not necessarily represent those of their affiliated organizations, or those of the publisher, the editors and the reviewers. Any product that may be evaluated in this article, or claim that may be made by its manufacturer, is not guaranteed or endorsed by the publisher.
